# Fifteen years of enzyme replacement therapy for mucopolysaccharidosis type VI (Maroteaux–Lamy syndrome): a case report

**DOI:** 10.1186/s13256-021-03240-3

**Published:** 2022-01-25

**Authors:** Isadora Andrade, River Ribeiro, Zumira A. Carneiro, Roberto Giugliani, Catarina Pereira, Claudia Cozma, Daniel Grinberg, Lluïsa Vilageliu, Charles M. Lourenco

**Affiliations:** 1 Faculdade de Medicina, Centro Universitário Estácio de Ribeirão Preto, Rua Abrahão Issa Halach, 980, Ribeirânia, Ribeirão Preto, SP 14096-160 Brazil; 2 Serviço de Genética Médica e Grupo de Pesquisa DR BRASIL, Hospital das Clínicas de Porto Alegre, Porto Alegre, RS Brazil; 3 Centogene AG, Rostock, Germany; 4 Departament de Genètica, Microbiologia i Estadística, Facultat de Biologia, Universitat de Barcelona, CIBERER, IBUB, IRSJD, Barcelona, Spain; 5 Departamento de Genética, Universidade Federal do Rio Grande do Sul, Porto Alegre, RS Brazil

**Keywords:** Mucopolysaccharidosis, MPS VI, Maroteaux–Lamy syndrome, Glycosaminoglycans, Arylsulfatase B, Enzyme replacement therapy

## Abstract

**Background:**

Mucopolysaccharidosis VI, or Maroteaux–Lamy disease, is an autosomal recessive disease characterized by deficiency of the enzyme arylsulfatase B in the lysosomal catabolism of glycosaminoglycans. Due to reduced (or even null) enzyme activity, glycosaminoglycans (mainly dermatan sulfate) accumulates, leading to a multisystemic disease. Mucopolysaccharidosis VI induces reduced growth, coarse face, audiovisual deficits, osteoarticular deformities, and cardiorespiratory issues, hampering the quality of life of the patient. Enzyme replacement therapy with galsulfase (Naglazyme, BioMarin Pharmaceuticals Inc., USA) is the specific treatment for this condition. Although studies have shown that enzyme replacement therapy slows the progression of the disease, the effects of long-term enzyme replacement therapy remain poorly understood.

**Case presentation:**

A 29-year-old, Caucasian, male patient diagnosed with mucopolysaccharidosis VI was treated with enzyme replacement therapy for over 15 years. Enzyme replacement therapy was initiated when patient was 13 years old. The patient evolved multiplex dysostosis, carpal tunnel syndrome, thickened mitral valve, and hearing and visual loss.

**Conclusions:**

Although enzyme replacement therapy did not prevent the main signs of mucopolysaccharidosis VI, it slowed their progression. Additionally, enzyme replacement therapy was associated with a longer survival compared with the untreated affected sibling. Taken together, the results indicate that enzyme replacement therapy positively modified the course of the disease.

## Background

Mucopolysaccharidoses (MPS) are a group of inborn errors of metabolism that are part of the lysosomal storage diseases (LSDs), and are classified into 11 main types (I, II, IIIA, IIIB, IIIC, IIID, IVA, IVB, VI, VII, and IX) in accordance with the deficient enzyme. Maroteaux–Lamy syndrome, also known as MPS VI, is due to the deficiency of the enzyme N-acetylgalactosamine 4-sulfatase (arylsulfatase B) [1, 2], which leads to the accumulation of glycosaminoglycans (GAGs) throughout the body, particularly dermatan sulfate. This condition, which was originally described by the French physicians Pierre Maroteaux and Maurice Lamy in 1963, is inherited in an autosomal recessive manner [3]. Although MPS VI is a rare disease, with an estimated incidence of 1.3–4.5 in 100,000 births [4], it is more frequent in some parts of the world, and there is a city in Brazil (Monte Santo, Bahia) where the incidence is nearly 1: 5000 live births [5]. Unfortunately, there are currently no regular newborn screening programs for MPS VI [6], although limited programs are in place in specific high-incidence areas [7].

The age of presentation and clinical manifestations of MPS VI may greatly vary. The spectrum of clinical phenotypes may include an increase in the anteroposterior diameter of the skull, coarse facial features, thick eyebrows, ocular proptosis, broad nasal bridge, thick lips, ogival palate, badly implanted teeth, macroglossia, short neck, narrow shoulders, globular abdomen, fingers and toes in a semi-flexion position, single palmar fold, short stature, dysostosis multiplex, generalized hypertrichosis, valgus knees, flat and thick feet, and less elastic skin [8, 9]. Despite the clear physical changes, the disease does not cause intellectual disability, unlike several other MPS types [10].

Cardiovascular lesions are frequent in MPS VI and constitute an important cause of morbidity and mortality [11, 12]. In fact, the deposition of GAGs in the myocardium and in blood vessels may result in progressive aortic and/or mitral valves degeneration, stenosis, and/or valvar insufficiency. The progression of these cardiac abnormalities may cause left ventricular hypertrophy and subsequent pulmonary congestion and heart failure [12].

Regarding the skeleton and the osteoarticular system, short stature and joint stiffness are the main manifestations of MPS VI. These symptoms are progressive and limiting due to the metaphyseal involvement and thickening and fibrosis of the joint capsule. Joint mobility is worse with increasing patient age. Thus, evaluation of mobility of the knees, hip, and elbows are considered a good marker of MPS VI evolution [13, 14]. Thoracolumbar kyphosis and oval vertebral bodies with inferior protrusion can also be verified in patients with MPS VI [13]. Wrists and hands may be affected by the enlargement and shortening of the metacarpals and phalanges, leading to flexure contracture of the fingers and a claw-hand aspect [1, 12].

Patients with MPS VI are more likely to present with glaucoma (50%) and progressive corneal opacification (95%) [6, 7, 12]. Abnormalities in the optic nerve may occur due to accumulation of GAGs in ganglion cells, compression of the optic nerve due to thickening of the dura mater and increased intracranial pressure. Alterations in the optic disc accompanied by mild to moderate edema has also been verified in 50% of patients with MPS VI [7, 12].

Not only the clinical manifestations may vary, but also the rate of progression of the disease, which may advance slowly, moderately, or rapidly. Within this context, the severe form commonly has its onset at the age of 2 years and a rapid progression is observed; the moderately and slow progressive forms are generally diagnosed later in adolescence or even in adulthood, having a more attenuated phenotype [6].

For many years, as MPS VI disease progressed, it was fatal to most of the patients. Hematopoietic stem cell transplantation (HSCT) became a therapeutic alternative to counteract the disease progression but is associated with a high risk of morbidity and mortality. The advent of enzyme replacement therapy (ERT), available since 2005, constituted a significant improvement in terms of therapy, changing the natural history of this illness [13]. The objective of this study is to report the clinical history of a patient with MPS VI treated with ERT for 15 years.

## Case presentation

A 29-year-old, Caucasian, male patient, born to nonconsanguineous parents, was referred for evaluation due to suspicion of being affected by an LSD as was his older brother in 1994, who presented a similar phenotype and has been investigated for MPS. The pregnancies were uneventful, and all prenatal evaluations were reported as normal. There was no exposure to teratogenic agents during pregnancy.

During infancy and childhood, there were no reports of delays in achieving motor skills. As the patient presented phenotypic traces of an LSD and his brother was diagnosed with MPS VI, he was also subjected to biochemical investigation for MPS (Table 1) and skeletal X-rays.

**Table 1:** Laboratory results

Urinary GAGs	235 μg GAGs/mg creatinine (NR: 13–45)
Activity of arylsulfatase B in leukocytes (colorimetric assay with artificial substrate)	6 nmol/hour/mg protein (NR: 72–176)
Activity of alpha-iduronidase in leukocytes (colorimetric assay with artificial substrate)	53 nmol/17 hour/mg protein (NR: 32–56)
Activity of iduronate sulfatase in plasma (colorimetric assay with artificial substrate)	292 nmol/4 hour/ml (NR: 122–463)
Molecular genetics analysis of the *ARSB* gene	Missense mutation c.944G > A (p.R315Q) in exon 5 (paternal inheritance) Frameshift mutation c.1534del23 in exon 8 (maternal inheritance)

*GAGs* glycosaminoglycans, *NR* normal range values

He received the confirmatory diagnosis of MPS VI at the age of 2.5 years (September 1994). Genotyping indicated compound heterozygosity for a missense mutation (p.R315Q9) and a frameshift mutation (c.1534del23) in the *ARSB* gene. As the phenotypic traces of MPS continued to progressively evolve, the patient was followed-up by a multidisciplinary team of healthcare specialists. The older brother died in 1995, at the age of 6 years, due to MPS-associated respiratory complications.

During clinical follow-up visits, the patient was physically examined to evaluate growth (weight, height, and cephalic perimeters), pulmonary and cardiac auscultation, mobility and gait, and organomegaly. The clinical progression of the disease was characterized by growth deficiency, joint contractures, and coarse facial features. In addition, abnormalities in cardiac valves, and conductive hearing loss and, due to chronic cranial hypertension, the patient developed bilateral optic atrophy and irreversible vision loss.

In October 2003, the patient was enrolled in a phase III clinical trial with ERT using a recombinant arylsulfatase B (galsulfase, Naglazyme, BioMarin Pharmaceuticals Inc., USA), and continued to receive weekly infusions of galsulfase, 1.0 mg/kg, when the study was completed.

In more than 15 years of ERT, the patient’s clinical condition was kept stable, with improvement of some parameters, such as hearing. Unfortunately, as his blindness was a consequence of optic nerve atrophy due to hydrocephalus, ERT failed to revert it.

At the age of 28 years, the patient displayed height below the third percentile, weight between the 75th and 90th percentiles, cephalic perimeter between the 50th and 95th percentiles, facial coarseness, depressed nasal bridge, thick lips, muscular contracture and joint stiffness, clawed hands, thick skull hair, umbilical hernia, hepatosplenomegaly, osteotendinous reflexes in upper and lower limbs grade 3, exhaustible clonus in lower limbs, and cutaneous-plantar reflex in extension.

Regarding the complementary exams, a radiographic study showed signs of dysostosis multiplex. Echocardiogram revealed a thickened mitral valve and electromyography showed the presence of multiple sites of nerve compression compatible with carpal tunnel syndrome. Finally, audiometry revealed conductive hearing loss.

Despite the important manifestations of the disease, the patient graduated in law, works regularly, and was taking classes in the business school, showing no intellectual deficit, as is expected in MPS VI. His survival is much longer compared with his untreated affected sibling and also to historical untreated controls [14].

## Discussion

As in most genetic conditions, there is no cure to MPS VI yet. Two therapeutic strategies have been employed to reduce disease manifestations and increase the quality of life of patients: HSCT and ERT. HSCT has rarely been employed in MPS VI, with a small case series reported. The results have shown that, in spite of an increase in arylsulfatase B activity, skeletal abnormalities were not corrected. In addition, the mortality risk associated to the treatment itself is nearly 30% [6, 7]. The infusion of the recombinant human enzyme arylsulfatase B has become the first line of pharmacological treatment for MPS VI since its first approval in 2005. Especially when initiated early, studies have shown a delay in the involvement of cardiovascular system, facial dimorphism, hepatomegaly, and physical development. Skeletal and ophthalmologic abnormalities, however, continue to progress [16–19].

There is growing evidence that early initiation of ERT in young patients has clear benefits in preventing the progression of the main disease manifestations [16–20]. In this regard, Horovitz and colleagues [16] described the effects of ERT for 6 years on four unrelated patients who started the therapy in their first year of life. These authors reported that many aspects of the condition were still present in the treated patients (as neurological, cardiac, and orthopedic manifestations) but with a reduced rate of progression, demonstrating that the age of ERT initiation is critical for a better prognosis. Corroborating this, Furujo and coworkers [17] demonstrated that 10 years of ERT in two Japanese siblings produced better results in the younger one, who initiated therapy at 6 months of age and did not display the typical symptoms of MPS VI (facial dysmorphism, hepatosplenomegaly, and auditory impairment), but skeletal changes such as the claw hands and opacity of the cornea progressed. These results reinforce the need of increasing awareness among the healthcare professionals to reduce the age of diagnosis, favoring the effects of ERT on MPS progression.

Although the patient was one of the first patients to initiate ERT in Brazil in 2003, he could not fully benefit from the treatment because he was 13 years old at the time and the progression of some clinical aspects of his condition were already considered irreversible, such as growth deficit, joint contractures, bilateral optic atrophy, and corneal opacity. Even in this limited scenario, the patient’s quality of life significantly improved, and he could recover much of his hearing ability. Despite dysostosis multiplex, the patient was able to graduate in law and has a relatively normal life with his family.

It has been reported that ERT may also improve endurance and respiratory capacity. Twenty-four weeks of ERT improved the performance in both 6- and 12-minute walk tests, as well as in the stair climbing test in patients with MPS VI aged between 6 and 22 years. Additional improvements in the 12-minute walk and in the stair climbing tests were obtained after 48 weeks of treatment. Joint pain and stiffness were also improved by 24-week ERT treatment [18]. Similarly, patient endurance was significantly improved by ERT. Before ERT, the patient was not able to stand or walk, using a wheelchair for locomotion. With ERT, the patient abandoned the wheelchair and can walk without support. The range of motion of the joints are still limited.

One of the most significant concerns of MPS VI is myelopathy of the spinal cord compression, which is potentially life-threatening and debilitating. ERT has not been successful in preventing cervical myelopathy; surgical decompression is required to achieve a satisfactory outcome and early surgery may be crucial [21]. Of note, detailed neurological evaluation should be performed in patients treated with ERT, especially in the period of improvement of the osteoarticular condition. The presence of neurological abnormalities (quadriparesis and/or spasticity, urinary retention secondary to neurogenic bladder, hyperreflexia with or without clonus, history of decreased tolerance to exercise or difficulty in getting up) should alert the physicians to a possible need for surgical decompression [21]. Currently, magnetic resonance imaging is considered the gold standard examination for the diagnosis of cervical cord compression. Surgical interventions may be challenging due to management of the airways and anesthesia during surgery [22]. In fact, clogged airways, chronic obstructive pulmonary disease, heart problems, and cervical instability may contribute to an increased risk of complications associated with anesthesia. Careful preoperation, anticipation of problems, and involvement of anesthesiologists with the physician accompanying the case may be critical to minimize the risk of preoperative mortality [22].

Improved quality of life and life expectancy of patients with MPS VI, as illustrated here, may draw attention to other disease-related complications, such as myelopathy of the spinal cord compression. In this regard, raising awareness among neurosurgeons, spine orthopedists, and anesthetists to MPS complications is fundamental to prevent complications during surgery.

## Conclusion

ERT slowed the progression of the cardinal clinical manifestation of the patient, who presented a longer survival in comparison with his untreated affected sibling who did not receive the treatment (not available at the time of the diagnosis). These results indicate that, even though ERT still does not represent the cure for MPS VI, this treatment has positively modified the natural course of the disease.

## Data Availability

The datasets analyzed during the current study are available from the corresponding author on reasonable request.

## Abbreviations

MPS VI: Mucopolysaccharidosis VI
ERT: Enzyme replacement therapy
GAGs: Glycosaminoglycans
HSCT: Hematopoietic stem cell transplantation
LSDs: Lysosomal storage diseases
